# Joint Flexibility and Isometric Strength Parameters Are Not Relevant Determinants for Countermovement Jump Performance

**DOI:** 10.3390/ijerph18052510

**Published:** 2021-03-03

**Authors:** Andreas Konrad, Marina Maren Reiner, Daniel Bernsteiner, Christoph Glashüttner, Sigrid Thaller, Markus Tilp

**Affiliations:** Institute of Human Movement Science, Sport and Health, University of Graz, Mozartgasse 14, A-8010 Graz, Austria; marina.reiner@uni-graz.at (M.M.R.); daniel.bernsteiner@edu.uni-graz.at (D.B.); christoph.glashuettner@edu.uni-graz.at (C.G.); sigrid.thaller@uni-graz.at (S.T.); markus.tilp@uni-graz.at (M.T.)

**Keywords:** jump performance, joint mobility, strength, maximum voluntary contraction (MVC), counter movement jump (CMJ)

## Abstract

Vertical jumps are of great importance as a performance predictor for many types of sports that require speed and agility. However, to date, it is not clear if flexibility and/or the strength of the different leg muscles are determinants for countermovement jump (CMJ) performance. Therefore, the purpose of this study was to relate isometric maximum voluntary contraction (MVC) torque and the flexibility of various muscle groups of the lower body with CMJ performance. Thirty-six healthy male volunteers participated in this study. The participants performed MVCs of the knee extensors, knee flexors, and plantar flexors on a dynamometer. Moreover, range of motion of the hip flexors and plantar flexors was assessed with 3D motion capture, and the range of motion of the knee flexors (hamstrings) was assessed with a Sit n’ Reach^®^ box. CMJs were assessed with a force platform. The correlation analysis revealed a significant moderate correlation of CMJ height with the flexibility of the hip flexors (rP = −0.39) and plantar flexors (rP = 0.47), but not the knee flexors. Moreover, we found that absolute MVC values are not related to CMJ height. However, we did find that knee extensor MVC relative to body mass is significantly related to CMJ height (rP = 0.33) with a moderate magnitude. Although we found significant correlations, the magnitudes of correlations vary between trivial and large according to a 90% confidence interval. Thus, this indicates that range of motion or strength of the assessed leg muscles can explain CMJ performance only to a limited extent.

## 1. Introduction

The ability to jump vertically is a predictor for performance in many types of sports (e.g., soccer [1]). Various factors such as force production, jump technique, flexibility of the affected joints, and anthropometrics are strong predictors of vertical jump performance [2]. Earlier studies investigated the contribution of the work of the leg joints during vertical jumps and reported different results. While Hubley and Wells [3] reported a major contribution of the knee joint (49%) and important contributions of the hip (28%) and ankle (23%) joints, Fukashiro and Komi [4] reported that the hip joint is the major contributor (51%; knee: 33%; ankle: 16%) in vertical jumps. A more recent study also found a major contribution of the hip joint in vertical jump height, in both good and poor vertical jump performers (43% and 41%, respectively) [5]. Vanezis and Lees [5] also concluded that strength and the rate of force development are better predictors for good jump performance than technique (e.g., with and without arm swing). Strength of the leg muscles was found to be a determinant parameter for vertical jump height in some [2,6] but not all studies [7]. Moreover, another study pointed out that strength is only related to vertical jump height when it is relative to body mass [8]. Besides strength, a further possible predictor might be the flexibility of the affected joints in a vertical jump. Godinho et al. [9] reported that ankle mobility (dorsiflexion) is positively related to countermovement jump (CMJ) performance. Assuming that greater flexibility leads to greater range of motion during the movement, this would imply a longer path for acceleration, and hence an increased take-off velocity [10]. However, further knowledge relating flexibility of the other leg muscles (e.g., knee and hip flexibility) as a possible predictor for CMJ performance is absent. There is evidence that a single stretching exercise of the knee flexors and hip flexors (including post stretching jumps) increases on the one hand the flexibility of these muscle tendon units and additionally on the other hand it can lead to an increase in CMJ performance [11]. Therefore, it can be assumed that a positive relation between flexibility of this muscle tendon units and CMJ performance exists. However, in summary, it is still unclear how strength and flexibility of the most relevant joints of the lower limbs (hip, knee, ankle) are related to vertical jump performance.

To date, to the best of our knowledge no study has investigated the impact of both isometric strength and flexibility of all the major joints of the lower leg on CMJ performance. Therefore, the purpose of this study was to relate the isometric strength and flexibility of the knee extensors, knee flexors, and plantar flexors with CMJ performance.

## 2. Materials and Methods

### 2.1. Participants

Godhino et al. [9] reported a correlation of 0.576 between CMJ and ankle mobility. Thus, in this study, to be safe, a correlation coefficient of 0.5 was used for the a priori sample size calculation. This revealed an optimal sample size of 29 subjects for our study (correlation: bivariate normal model, pH1 = 0.5, α = 0.05, β = 0.80). Therefore, to meet this requirement, 36 healthy non-professional male athletes (age: 23.64 ± 4.11 years; body mass: 81.2 ± 6.8 kg; height: 181.8 ± 5.2 cm) were recruited to participate in this study. Participants with a history of lower leg injuries, any type of neuromuscular disorder, and elite athletes were excluded from the study. Participants were asked to be in a rested state and not to perform strenuous workouts in the 72 h prior to the measurements. The participants signed a written informed consent form, and ethical approval was obtained by the local ethics commission of the University of Graz. The study was performed in accordance with the Declaration of Helsinki.

### 2.2. Experimental Design

Participants were asked to visit the laboratory on two separate occasions. The first visit was a familiarization session to explain the test procedure and familiarize the participants with the equipment and the test procedures. The second visit was the test session for data acquisition. On the second visit, participants performed a 5-min warm-up on a stationary bike (Monark, Ergomedic 874 E, Sweden) at 60 rev/min. After the warm-up, the isometric maximum voluntary contraction (MVC) of three different leg muscle groups (knee extensors, knee flexors, plantar flexors) was recorded on a dynamometer. Surface electromyography (EMG) was recorded from the vastus lateralis, semimembranosus, and gastrocnemius medialis during the MVC measurements. Moreover, passive range of motion (RoM) tests of the hip flexors (modified Thomas test) and plantar flexors (standing wall pushes) were undertaken with a 3D motion capture system. Passive knee flexor (hamstring) RoM was tested with the Sit n’ Reach^®^ test. As the last measurement, countermovement jump performance was assessed on a force platform.

### 2.3. Measures

#### 2.3.1. Maximum Voluntary Contraction (MVC) Tests

After the standardized warm-up, the MVC tests for the knee extensors, knee flexors, and plantar flexors of the dominant leg were performed on an isokinetic dynamometer (CON-TREX^®^ Multijont, Duebendorf, Switzerland). For the knee extensor MVC, the participant was seated on the dynamometer with both hip and knee angles of 110° ([12]; 180° = anatomical zero [13]). For the knee flexor MVC, the participant was positioned supine on the dynamometer with hip and knee angles of 90° and 110°, respectively. For the plantar flexor MVC, the participant lay prone on the dynamometer and the ankle angle was set to 90° ([14]; 90° = anatomical zero).

A laser was used to align the center of rotation at the dynamometer and the joint axis of the knee and ankle. In each position, the trunk and test leg were fixed with straps to minimize evasive movement. Three isometric MVCs of 5 s each, with a 1-min rest in between each attempt, were performed for each muscle group. The participant was instructed to push as hard as possible. The attempt with the highest torque value was taken for further analysis.

#### 2.3.2. Range of Motion (RoM) Tests

The RoM of the plantar flexors and hip flexors was tested with an eight-camera 3D motion capture system (Qualisys, Göteborg, Sweden) with 100 frames per second. System calibration was performed at the beginning of each measurement day. Reflective markers of 1 cm in diameter were placed according to the Qualisys Gait module (type: cast) on the participant’s hip (with two extra markers on the iliac crest to ensure a proper measurement in a supine position) and test leg. Six markers were placed near the pelvis, two on the knee, and six on the lower limb. In addition, clusters of four markers were placed on the shank and on the thigh. To test ankle RoM, a standing wall push [14] was performed three times for 5 s each. The participant stood upright in front of a wall, with both hands on the wall at chest height. After the start command, the test leg was moved behind the body. The participant was then asked to move the test leg with an extended knee as far as possible behind the body, with the heel touching the ground, to achieve a stretch until the point of discomfort. Moreover, the RoM of the hip flexors was tested with a modified Thomas test [15]. The participant was asked to perform three modified Thomas tests of the dominant leg for 5 s each on a medical treatment bed. The participant lay supine, with the iliac crest close to the edge of the bed [15], hips and knees at 90°, and knees fixed by hands with extended arms to ensure the same hip angle at the initial position between the measurements and a flat lumbar spine. The legs were completely relaxed. While holding the contralateral leg in position, the test leg was lowered unassisted toward the floor and the participant was asked to relax in the end position. For the analysis, the mean of the two most similar attempts was calculated. Furthermore, to test the RoM of the knee flexors (hamstring muscles), the participant was asked to perform three bilateral Sit n’ Reach^®^ tests with extended knee joints. A Sit n’ Reach^®^ box (Fabrication Enterprises, Model-number: 12-1086; White Plains, New York, NY, USA) was used to measure the knee flexor RoM. The starting position was a sitting position with extended legs and an upright upper body, and the arms were held parallel to the ground in front of the trunk. The participant was asked to shift the spacer along the measuring box as far away as possible in the direction of the toes, with extended knees and arms. The end position of the spacer was noted and the best attempt out of three was used for further analysis.

#### 2.3.3. Countermovement Jumps (CMJs)

For the dynamic strength measurements, the participant was asked to perform three CMJs, with a 1-min rest in between, on a transportable force platform (Quattro Jump, Kistler GmbH, Winterthur, Switzerland, 500 Hz recording frequency). Start and end position was upright, in a hip-wide standing position, with hands placed on the hips [16]. The hands were not allowed to be moved during the whole measurement, in order not to influence the jump height [17]. On command, the participant lowered their center of mass by bending the knees to a self-selected grade, and immediately after reaching the lowest position, they jumped vertically as high as possible. The highest jump height was used for the further analysis.

### 2.4. Statistical Analyses

SPSS (version 25.0, SPSS Inc., Chicago, IL, USA) was used for all the statistical analyses. A Kolmogorov–Smirnov test was used to verify the normal distribution of all the variables. A Pearson’s correlation analysis (reported as rP) was used to calculate the relationship between the variables. The alpha level was set to 0.05. The effect sizes of rP were established following the suggestions of Hopkins [18]. Thus, the value of the effect size is the same as the correlation. An effect size of 0–0.1, 0.1–0.3, 0.3–0.5, 0.5–0.7, 0.7–0.9, and 0.9–1 was defined as a trivial, small, moderate, large, very large, nearly perfect or perfect, respectively. 

## 3. Results

### 3.1. Correlation Analysis of Countermovement Jump Performance with Range of Motion of the Lower Leg Muscles

**Figure 1:**
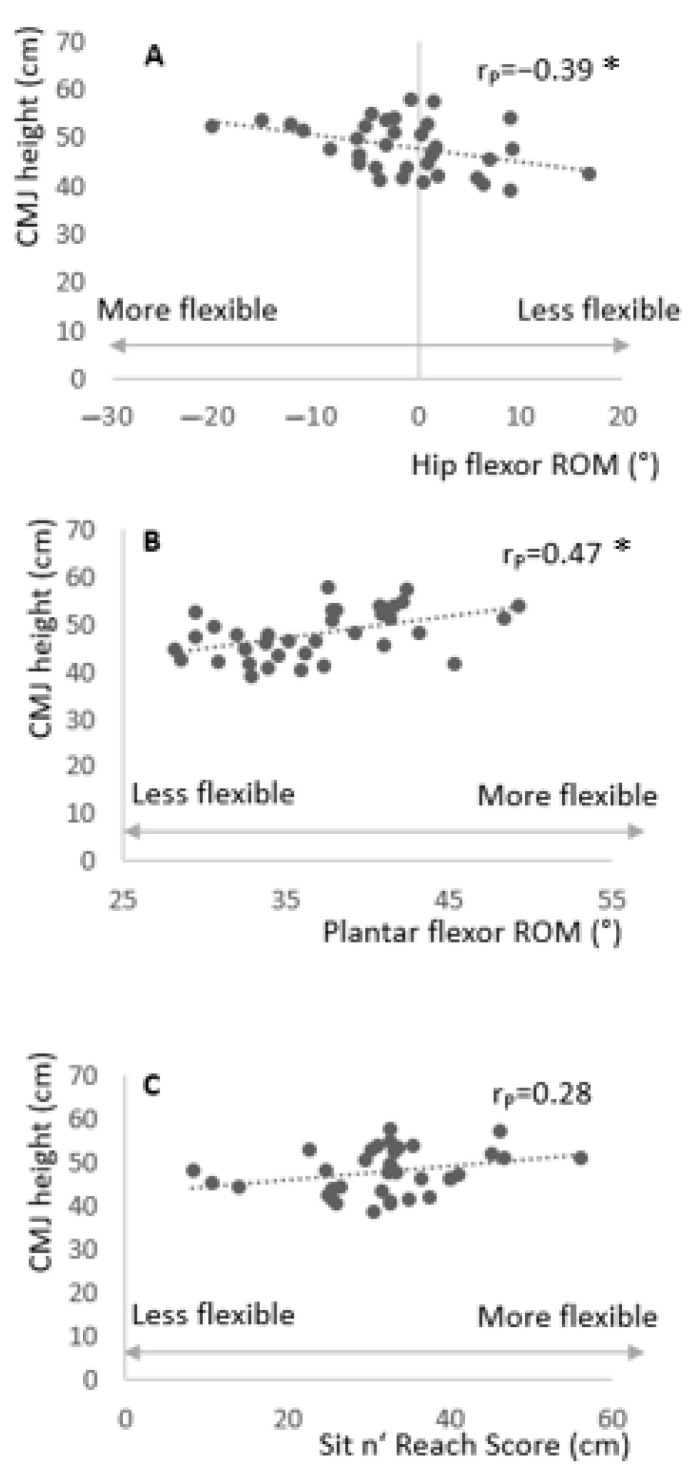
Correlation of countermovement jump (CMJ) height with hip flexor range of motion (RoM) (**A**), plantar flexor RoM (**B**), and Sit n’ Reach^®^ score (**C**). * = indicates a significant correlation.

The correlation analysis revealed a significant moderate correlation between CMJ performance and both hip flexor RoM (rP = −0.39; P = 0.02; CI (90%): −0.13 to −0.60) and plantar flexor RoM (rP = 0.47; P = 0.004; CI (90%): 0.22 to 0.66). The 90% confidence interval revealed a range of both correlations between small to large. Moreover, there was no significant correlation between CMJ performance and knee flexor RoM (rP = 0.28; P = 0.09; CI (90%): 0.00 to 0.52) (see Figure 1).

### 3.2. Correlation Analysis of Countermovement Jump Performance with Maximum Isometric Voluntary Contraction Torque of the Lower Leg Muscles

**Figure 2:**
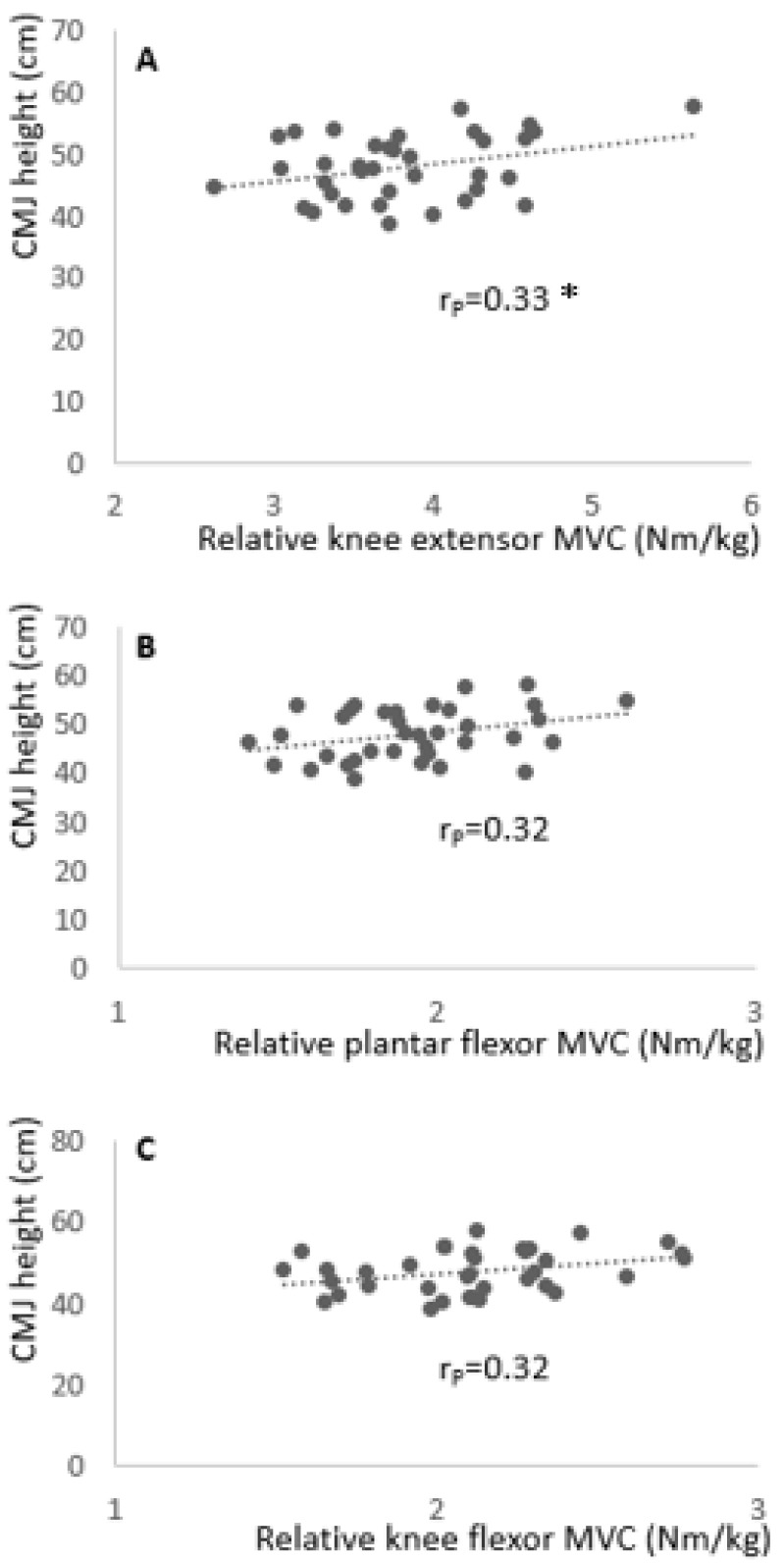
Correlation of CMJ height with relative knee extensor maximum voluntary contraction (MVC) (**A**), relative plantar flexor MVC (**B**), and relative knee flexor MVC (**C**). * = indicates a significant correlation.

The correlation analysis revealed no significant correlation between CMJ performance and knee extensor MVC (rP = 0.20; P = 0.23; CI (90%): −0.08 to 0.45), plantar flexor MVC (rP = 0.15; P = 0.38; CI (90%): −0.13 to 0.41), and knee flexor MVC (rP = 0.18; P = 0.30; CI (90%): −0.10 to 0.44). Moreover, the correlation analysis revealed a significant moderate correlation between CMJ performance and relative (to body mass) knee extensor MVC (rP = 0.33; P = 0.047; CI (90%): 0.06 to 0.56), with a range of correlation between trivial and large according to the 90% confidence interval. Moreover, there was no significant correlation between CMJ performance and relative plantar flexor MVC (rP = 0.32; P = 0.06; CI (90%): 0.05 to 0.55) or relative knee flexor MVC (rP = 0.32; P = 0.06; CI (90%): 0.05 to 0.55) (see Figure 2).

## 4. Discussion

The purpose of this study was to investigate if a relationship between isometric strength or flexibility of the relevant leg muscles with CMJ performance exists. Pearson correlation coefficients showed a significant linear relationship between both hip flexor RoM and plantar flexor RoM with CMJ height. However, these correlations showed a moderate magnitude only and can be considered as of minor relevance. Moreover, no significant relationship was observed for the knee flexor muscles with CMJ height. Furthermore, a significant positive correlation was observed between CMJ height and knee extensor isometric MVC, but only when normalized to body mass and with a moderate magnitude. Hence, this relationship also appears to be of minor relevance. Plantar flexor and knee flexor MVC seem not to be related to CMJ height.

A positive correlation between plantar flexor RoM and CMJ height was observed by Godinho et al. [9]. Although significant, the results of our study showed only minor correlations between plantar flexor RoM and CMJ height (rP = 0.47; P = 0.004; CI (90%): 0.22 to 0.66). Additionally, the present study provides only weak evidence that greater hip flexor RoM is related to CMJ height (rP = −0.39; P = 0.02; CI (90%): −0.13 to −0.60). Leg extensor (hamstring) flexibility seems not to be related to CMJ height (rP = 0.28; CI (90%): 0.00 to 0.52, P = 0.09). Although the present study revealed significant correlations between both hip flexor RoM and plantar flexor RoM with CMJ height, the magnitude of the correlations was only moderate in both cases. Consequently, the explained variance of the relation between hip flexor RoM, ankle RoM and CMJ height was only 15.2% and 22.1%, respectively, with confidence intervals ranging from small to large which indeed can be interpreted as poor evidence [19]. This suggests that other factors (e.g., joint power, dynamic strength) are more closely related to CMJ height and explain the remaining variance.

A greater RoM in the lower limb joints could lead to greater movement amplitudes in the counter movement phase of a jump. As the depth of the counter movement was not specified, we assumed that our participants chose their squat depth according to their RoM and the comfortable tension in their muscles and tendons, as the maximum RoM would lead to pain [20]. The idea that more flexible subjects might use deeper squats, prolonging the path of acceleration in the upward movement, could not be confirmed by our further analysis. We found no significant difference with an unpaired t-test (P = 0.37) in the squat depth of the counter movement between the more flexible participants (37.1 ± 4.6 cm) and the less flexible participants (35.4 ± 6.0 cm). 

Besides the flexibility parameters related to CMJ height, we also conducted correlations between strength parameters and CMJ height. Despite the observed significant positive correlations between knee extensor MVC and CMJ (rP = 0.33; P = 0.047), these provide only weak evidence because of its uncertain magnitude which ranged from trivial to large (CI (90%): 0.06 to 0.56). Knee extensor MVC (related to body mass) only explained 10.9% of the variance in CMJ height which limits its importance [19]. This goes in line with findings by Young et al. [7], who reported no correlations between jumping performance and strength parameters. However, Krizaj et al. [21] reported knee extensor strength as an important predictor for jump capacity in elite soccer players [21]. Vanezis and Lees [5] concluded that a better jump performance was associated with the strength and rate of force development in all the relevant joints and muscles. Though no relationships between relative knee flexor MVC (P = 0.06; post-hoc power 0.52) and relative plantar flexor MVC (P= 0.06; post-hoc power 0.52) with CMJ height were observed in this study. When not relative to body mass, isometric MVC showed no significant relationship with CMJ height in all three tested muscle groups (knee extensors rP = 0.20 (CI (90%): −0.08 to 0.45); plantar flexors rP = 0.15 (CI (90%): −0.13 to 0.41); knee flexors rP = 0.18 (CI (90%): −0.10 to 0.44)). A reason for the low and less meaningful correlations between the relative strength and CMJ height could be that isometric strength (isometric MVC) at one specific angle is not closely related to a dynamic movement (CMJ). Future studies should therefore assess other strength and power parameters (isokinetic torque, isometric torque at different angles, but also the rate of force development, etc.) and relate them to CMJ height. Moreover, various strength and power parameters from the hip joint should be related to CMJ height in future studies as this was shown to be an important predictor for vertical jump height [22].

A limitation of this study was the moderate magnitude of correlations between CMJ height to hip flexor RoM (rP = −0.39; P = 0.02; CI (90%): −0.13 to −0.60), plantar flexor RoM (rP = 0.47; P = 0.004; CI (90%): 0.22 to 0.66), and relative knee extensor MVC (rP = 0.33; P = 0.047; CI (90%): 0.06 to 0.56), respectively. Hence, the variance explained by hip flexor RoM, plantar flexor RoM, and relative knee extensor MVC in CMJ height was only 15.2%, 22.1%, and 10.9%, respectively. Additionally, the relative wide range of the 90% confidence interval (trivial to large) especially in the correlation between CMJ performance and relative knee extensor MVC can be seen as a further limitation [19]. Thus, caution has to be taken not to overemphasize these results. However, future intervention studies should test the causality of our findings by testing, e.g., if RoM increasing stimuli (e.g., stretching training) applied by the hip flexor or ankle joint can increase CMJ height.

## 5. Conclusions

Although we found significant correlations, the magnitudes of correlations vary between trivial and large according to a 90% confidence interval. Thus, this indicates that RoM or isomeric strength of the assessed leg muscles can explain CMJ performance only to a limited extent. Therefore, we recommend that future studies should investigate the relationship of dynamic strength parameters in the same and other muscle groups (e.g., around the hip joint).

## Data Availability

All the data assessed within this study are presented in the manuscript.
